# Initial amplification of the HPV18 genome proceeds via two distinct replication mechanisms

**DOI:** 10.1038/srep15952

**Published:** 2015-11-02

**Authors:** Marit Orav, Jelizaveta Geimanen, Eva-Maria Sepp, Liisi Henno, Ene Ustav, Mart Ustav

**Affiliations:** 1 University of Tartu, Institute of Technology, Nooruse 1, 50411 Tartu, Estonia; 2 Estonian Biocentre, Riia 23, 51010 Tartu, Estonia; 3 Icosagen Cell Factory OÜ, Eerika tee 1, 61713, Õssu, Estonia; 4 Estonian Academy of Sciences, Kohtu 6, 10130 Tallinn, Estonia

## Abstract

Determining the mechanism of HPV18 replication is paramount for identifying possible drug targets against HPV infection. We used two-dimensional and three-dimensional gel electrophoresis techniques to identify replication intermediates arising during the initial amplification of HPV18 episomal genomes. We determined that the first rounds of HPV18 replication proceed via bidirectional theta structures; however, a notable accumulation of almost fully replicated HPV18 genomes indicates difficulties with the completion of theta replication. We also observed intermediates that were created by a second replication mechanism during the initial amplification of HPV18 genomes. The second replication mechanism does not utilize specific initiation or termination sequences and proceeds via a unidirectional replication fork. We suggest a significant role for the second replication mechanism during the initial replication of the HPV18 genome and propose that the second replication mechanism is recombination-dependent replication.

Human papillomaviruses (HPVs) are small dsDNA viruses with approximately 8-kbp circular genomes. These important pathogens infect the keratinocytes of stratified cutaneous or mucosal epithelia, causing mainly benign hyperplastic lesions. To date, 170 different types of HPVs have been identified^1^. The mucosal viruses can be divided into high- and low-risk types. Lesions caused by high-risk HPV types, including types 16, 18, 31 and 45, have the potential for malignant progression, whereas low-risk HPV types do not display such a propensity^2^. HPV infections are mainly associated with cervical cancer^3^,^4^; however, the virus has also been implicated in the development of other anogenital^5^ and head and neck cancers^6^.

HPV genomes replicate in the host cell as nuclear multicopy extrachromosomal episomes. The HPV genomes undergo three separate phases of replication. The first phase begins shortly after infection and results in a rapid increase in viral genome copy number^7^. Initial papillomavirus replication is triggered and modulated by the viral replication factors E1 and E2^8^,^9^,^10^,^11^,^12^,^13^, which direct the cellular replication machinery to the replication origin situated in the non-coding region of the viral genome^14^,^15^. The initial amplification phase is followed by a stable maintenance of the viral episomes. It is unknown which replication mechanism is behind the transient amplification phase; however, the stable maintenance replication of papillomavirus genomes proceeds via bidirectional theta structures^16^,^17^. The last stage of the papillomavirus replication cycle is characterized by a vegetative amplification of the viral genomes^7^. Both bidirectional theta replication^18^ and rolling-circle replication^16^ have been suggested as the mechanisms for the late amplification phase of viral genomes.

In the present study, we used the U2OS-based model system^19^ to study replication intermediates (RIs) that arise during the initial amplification of the wild-type HPV18 (HPV18wt) and HPV18E8^−^ mutant genomes. Eliminating the expression of the E8/E2 protein, which is a repressor of transcription and replication, enhances the replication capability of several HPV types^20^,^21^,^22^,^23^. The HPV18E8^−^ mutant has been previously described^24^,^25^,^26^,^27^ and displays up to a hundred-fold more efficient replication than the wild-type genome^28^; however, the intermediates that arise during the replication of the HPV18wt and E8^−^ genomes are identical, making the E8^−^ mutant genome a useful tool for the analysis of RIs. The HPV18 RIs were analyzed via two-dimensional neutral/neutral (2D N/N), two-dimensional neutral/alkaline (2D N/A) and three-dimensional neutral/neutral/alkaline (3D N/N/A) agarose gel electrophoresis (AGE) followed by Southern blotting. We also analyzed RIs that arose during the initial amplification of HPV18E8^−^ mutant genomes in HaCaT cells and found them to be similar to the RIs that arose during the initial amplification of HPV18 genomes in the U2OS cell line.

Our results demonstrate that the initial amplification replication of HPV18 genomes proceeds via bidirectional theta structures. Bidirectional theta replication is initiated at the origin of replication, which is situated in the non-coding region of the HPV genome^13^, and results in the accumulation of almost fully replicated, late theta intermediates. HPV18 replication also results in intermediates that are not characteristic of bidirectional theta replication. These additional RIs are most compatible with a unidirectional mode of replication that does not have a specific initiation or termination site. We propose that the second mechanism involved in the amplification of HPV18 genomes may be recombination-dependent replication.

## Results

### 2D N/N AGE analysis of uncut RIs created during the initial replication of episomal HPV18 genomes in U2OS cells

**Figure 1:**
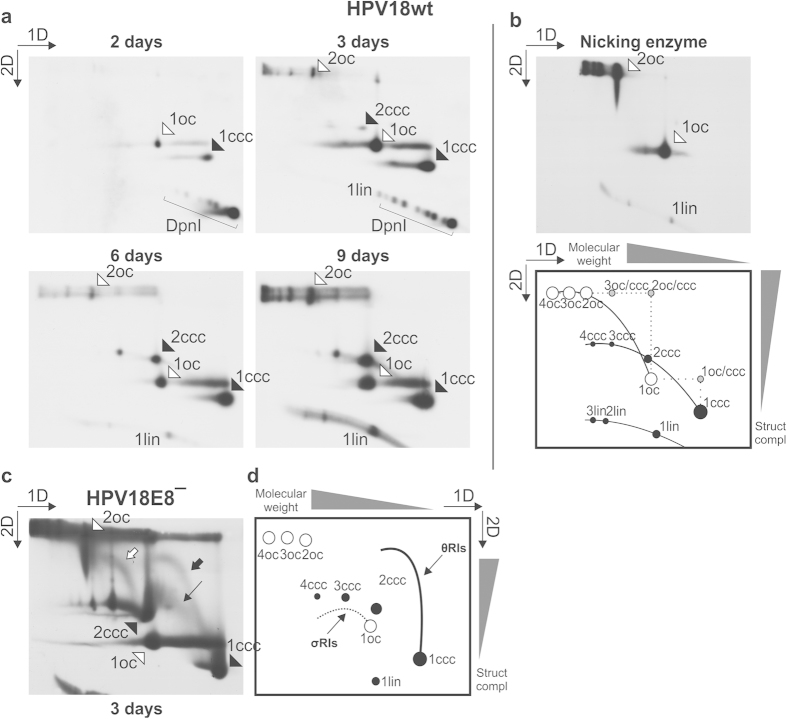
2D N/N AGE analysis of uncut HPV18wt episomes. oc, open circular molecules; ccc, covalently closed circular molecules; lin, linear molecules; DpnI, the fragments of DpnI-digested unreplicated genomes. Black arrowheads mark the HPV18 genomes in ccc form, and white arrowheads mark the HPV18 genomes in oc form. The numbers before the abbreviations denote the number of monomeric genome copies within the molecule. The direction of the gel electrophoresis in the first (1D) and second (2D) dimension is indicated in the top left (**a–c**) or right (**d**) corner throughout the panel. (**a**) 2D N/N AGE analysis of DpnI-treated, uncut HPV18wt episomes extracted from U2OS cells 2 to 9 days post-transfection. (**b**) 2D N/N AGE analysis of the nicking enzyme Nb.Mva1269I-treated HPV18wt episomes extracted from U2OS cells 5 days post-transfection and a schematic depiction of the expected migration patterns of circular molecules following 2D N/N AGE^31^,^39^. The direction for the increase of molecular weight and structural complexity is indicated along the top and right edges of the scheme. **(c)** 2D N/N AGE analysis of uncut RIs arising during the initial amplification replication of HPV18E8ˉ episomes. Bold arrows, putative theta RIs; thin arrow, diffuse putative RIs. **(d)** A schematic depiction of the expected migration patterns of bidirectional theta (θRIs) and rolling-circle RIs (σRIs)^31^,^39^. The direction for the increase of molecular weight and structural complexity is indicated along the top and right edges of the scheme.

**Figure 2:**
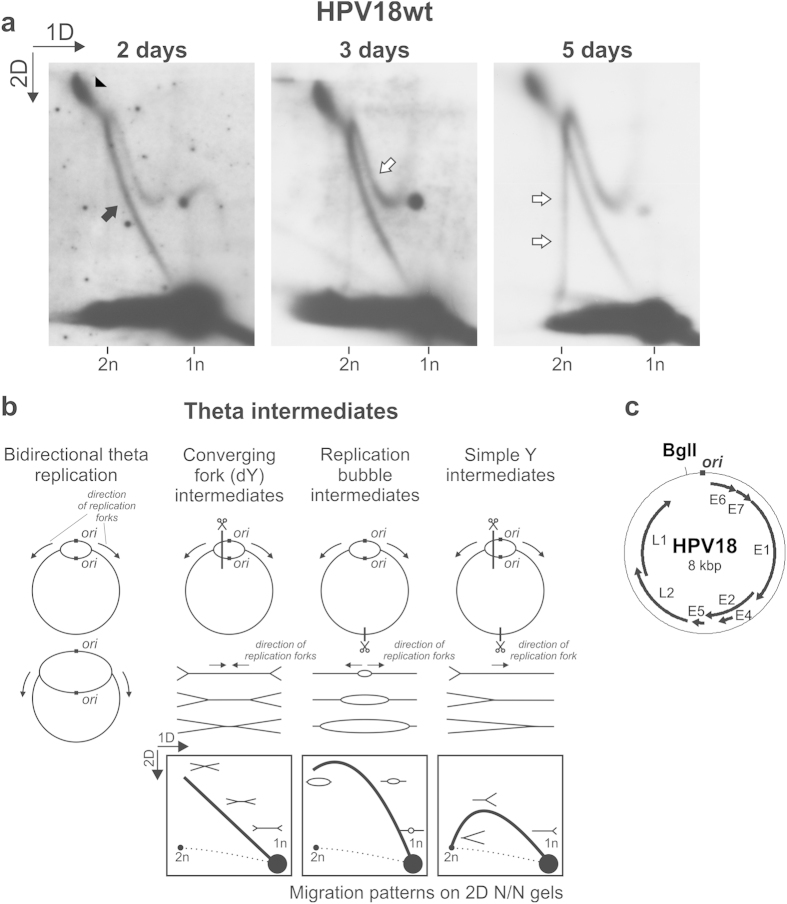
Analysis of linearized RIs arising from the HPV18wt genome. (**a**) 2D N/N AGE of BglI-digested HPV18wt episomes extracted from U2OS cells 2, 3 or 5 days post-transfection. 1n, monomeric (8-kbp) linear molecules; 2n, dimeric (16-kbp) linear molecules. Black arrowhead, putative almost fully replicated, late theta RIs; black arrow, dY RIs arising via bidirectional theta replication; white arrows, non-theta molecules. The direction of the gel electrophoresis in the first (1D) and second (2D) dimension is indicated in the top left corner of the panel. (**b**) A schematic overview of possible RIs created following the digestion of circular molecules undergoing bidirectional theta replication and their migration patterns during 2D N/N AGE^39^. The approximate position of the origin of bidirectional theta replication is marked as *ori*^13^. (**c**) The position of the BglI restriction site in the HPV18wt genome with regard to the origin of bidirectional theta replication (*ori*).

HPV18wt genomes, produced as covalently closed circular minicircles^29^, were transfected into U2OS cells by electroporation. Low molecular weight (LMW) DNA was extracted at 2, 3, 6 and 9 days post-transfection, purified^30^, and digested with DpnI to remove input HPV genomes. The undigested, replicated HPV18wt genomes were analyzed using 2D N/N AGE (Fig. 1a). Circular molecules can take either open circular (oc) or covalently closed circular (ccc) topological forms. The migration patterns for oc and ccc molecules were determined by treating HPV18 LMW DNA samples with the nicking enzyme Nb.Mva1269I (Fig. 1b), which converts ccc HPV genomes into oc molecules, thus enabling identification of these two topological forms (compare Fig. 1a to 1b). A scheme depicting the theoretical mobility of different circular forms of extrachromosomal HPV18 during 2D N/N AGE is also presented in Fig. 1b^31^. Two days post-transfection, the HPV18wt genomes synthesized in U2OS cells were mainly in the form of oc and ccc monomers (Fig. 1a, 2 days, marked by white and black arrowheads, respectively). The fragments of DpnI-digested unreplicated HPV18 genomes appeared on the arc of linear DNA below the 1lin, 1ccc and 1oc molecules (Fig. 1a, 2 and 3 days, DpnI). The oligomeric forms of HPV18 genomes appeared over time (Fig. 1a, 3–9 days). We have previously demonstrated the increasing prevalence of oligomeric HPV genomes in HPV-transfected U2OS cells in time^19^,^26^ and characterized these oligomers as episomal head-to-tail concatemeric molecules, which likely arise from monomeric HPV genomes through replication-dependent homologous recombination^26^. HPV oligomeric episomes have also been detected in clinical samples obtained from HPV-associated cervical lesions^32^,^33^,^34^,^35^,^36^ and HPV-immortalized human keratinocytes^37^,^38^. The present results (Fig. 1a) indicate the accumulation of monomeric and oligomeric episomal HPV18 genomes in U2OS cells, even after a 9-day cultivation period.

To identify RIs and replication products that arise during the first phase of HPV18 replication, we took advantage of the increased replication capability provided by the elimination of E8/E2 protein expression from the viral genome. Uncut LMW DNA extracted from HPV18E8^−^-transfected U2OS cells 3 days post-transfection was analyzed via 2D N/N AGE (Fig. 1c). The expected migration patterns of the monomeric and oligomeric circular plasmids and theta and rolling-circle RIs are shown on the scheme depicted in Fig. 1d 31,39. Signals that are similar to theta RIs are clearly visible during the analysis (Fig. 1c, marked by bold arrows). One of these signals emanates from the 1ccc molecule (Fig. 1c, black arrow). The origin of the second signal (Fig. 1c, white arrow), however, is less clear and could represent RIs arising from either the 1oc or 2ccc molecule. In addition to the signals that possess clear similarities with intermediates of theta replication, there is also a signal that displays characteristics common to RIs (increasing molecular weight coupled with changes in topological complexity) but differs from the theta RIs (Fig. 1c, marked by a thin arrow). This diffuse signal is visible between the 1ccc and 2ccc molecules and is as of yet unidentified in origin.

The rolling-circle RIs (also referred to as sigma RIs) result in the so-called eyebrow-shaped signal during 2D N/N AGE (Fig. 1d, σRIs)^31^,^40^. No such signal was observed during the 2D N/N AGE analysis of the uncut HPV18 genomes (Fig. 1c).

The 2D N/N AGE of uncut HPV18E8^−^ RIs indicates that during the initial E1 and E2-dependent phase of viral replication, signals resembling uncut theta RIs can be detected. In addition to theta RIs, a signal representing as of yet unidentified RIs was also detected. Notably, all RIs originated from extrachromosomal HPV18 genomes, and there was no indication of the involvement of rolling-circle replication in HPV replication.

### Two different populations of molecules arise during the initial amplification of the HPV18wt genome

Circular molecules undergoing bidirectional theta replication contain two replication forks that initiate at the origin of replication and then progress in opposite directions. If these circular replicating molecules are digested with a restriction enzyme, the position of the restriction site relative to the position of the origin of replication determines the structure of the resulting digested RIs. In principle, circular molecules replicating via theta structures can be converted into three different types of RIs: converging fork intermediates (also known as the double Y or dY intermediates), replication bubble intermediates and simple Y RIs (Fig. 2b). Because of the considerably different shapes of all three types of RIs, each creates a unique migration pattern that can be detected via 2D N/N AGE (Fig. 2b)^39^.

HPV18wt LMW DNA samples extracted 2, 3 or 5 days post-transfection were digested with BglI, which cleaves the HPV18 genome near the origin of replication (*ori*) situated in the non-coding region of the viral genome (Fig. 2c)^13^. If the initial HPV genomic amplification occurred via bidirectional theta replication, the 2D N/N analysis of HPV18 RIs should reveal the presence of the signal characteristic of dY intermediates (Fig. 2b). Two days post-transfection, such a signal was clearly detectable (Fig. 2a, 2 days, marked by a black arrow). We also observed a notable accumulation of molecules with branched shape and large molecular mass (Fig. 2a, 2 days, marked with a black arrowhead). Because these large and branched molecules migrate at the end of the signal representing the dY intermediates, they may represent nearly fully replicated, late theta RIs.

Three days post-transfection, an additional signal appeared with similar prominence to the dY intermediates (Fig. 2a, 3 days, marked by a white arrow); five days post-transfection, signals representing molecules with remarkable structural variability and molecular mass similar to that of dimeric HPV genomes (16 kbp) appeared (Fig. 2a, 5 days, marked by two white arrows). These novel signals represent molecules that could not arise during the bidirectional theta replication of monomeric HPV genomes. The analysis of uncut HPV18E8^−^ genomes indicated that bidirectional theta replication might also be initiated from dimeric HPV genomes (Fig. 1c, marked with a white arrow). Following BglI digestion, the bidirectional theta replication of dimeric HPV genomes with only one active origin of replication would have resulted in simple Y RIs (in principle similar to Fig. 2b, simple Y intermediates); however, the migration pattern of these novel molecules did not resemble the migration pattern of simple Y molecules (compare Fig. 2a to 2b). It must be noted that the migration pattern of these novel molecules with regard to the migration pattern of theta RIs did not change upon altering the conditions used for 2D N/N AGE (Supplementary Fig. 1S). We therefore propose that these signals mark the presence of molecules that are not connected to bidirectional theta replication.

Our data indicate that two different groups of molecules arise during the initial amplification of the HPV18wt genome. Intermediates arising via bidirectional theta replication are initially prevalent in the U2OS cells, indicating a significant role for theta replication in the early amplification of the HPV genome. The accumulation of large, branched RIs may indicate difficulties with the completion of bidirectional theta replication and the separation of created daughter molecules. Over time, however, a novel set of molecules becomes as prevalent as theta RIs. The analysis of linearized RIs also suggests that the signal resembling uncut bidirectional theta replication intermediates observed in Fig. 1c (marked with a white arrow) does not originate from dimeric HPV genomes. However, these results do not rule out the possibility that HPV dimers could be used for the initiation of bidirectional theta replication at a lower efficiency compared with the monomeric genomes.

### HPV18 initial amplification results in RIs created via two distinct replication mechanisms

**Figure 3:**
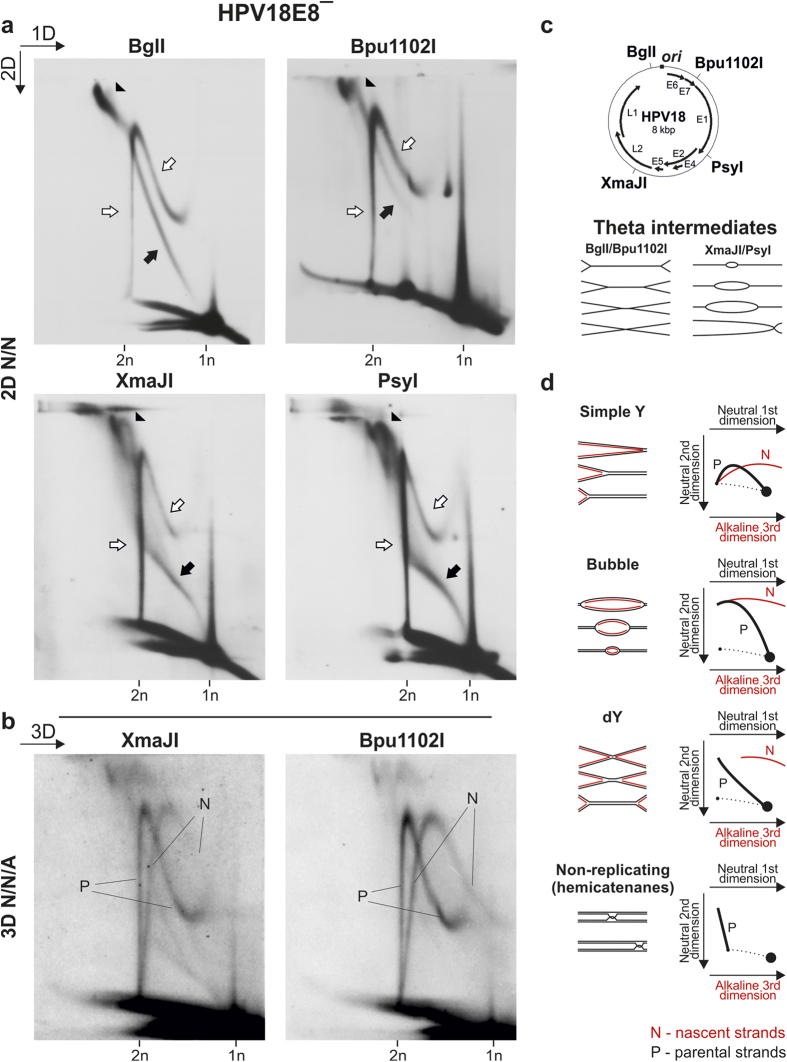
Analysis of HPV18E8ˉ RIs linearized with four different restriction enzymes. 1n, monomeric (8-kbp) linear molecules; 2n, dimeric (16-kbp) linear molecules. (**a**) 2D N/N AGE analysis of episomal HPV18E8ˉ RIs extracted from U2OS cells 3 days post-transfection and linearized with BglI, Bpu1102I, XmaJI, and PsyI. Black arrowheads, putative almost fully replicated, late theta RIs; black arrows, theta RIs; white arrows, non-theta molecules. The direction of the gel electrophoresis in the first (1D) and second (2D) dimension is indicated in the top left corner of the panel. (**b**) 3D N/N/A AGE analysis of XmaJI- and Bpu1102I-digested HPV18E8ˉ episomes extracted from U2OS cells 5 days post-transfection. P, parental strands; N, nascent strands. The direction of the gel electrophoresis in the third dimension (3D) is indicated in the top left corner. (**c**) The position of the BglI, Bpu1102I, XmaJI and PsyI enzyme recognition sites in the HPV18 genome (*ori* marks the approximate position of the origin of bidirectional theta replication^13^) and a scheme depicting bidirectional theta RIs digested with BglI/Bpu1102I and XmaJI/PsyI. (**d**) Migration patterns of nascent and parental strands originating from different types of RIs and parental strands of non-replicating molecules (represented by hemicatenanes) during 3D N/N/A AGE analysis^39^,^41^.

To further analyze the molecules that are present during the initial amplification of HPV genomes, LMW DNA extracted from HPV18E8^−^-transfected U2OS cells 3 days post-transfection was linearized with either BglI, Bpu1102I, XmaJI or PsyI and analyzed via 2D N/N AGE (Fig. 3a). The restriction enzymes were selected based on the positions of their cleavage site relative to the position of the origin of replication (*ori*) situated in the non-coding area of the HPV18 genome^13^ (Fig. 3c). The HPV18E8^−^ mutant genome was selected for this and all following analyses because of its increased replication capability. Similar RIs were present during the replication of the HPV18wt and E8^−^ genome (compare Fig. 2a with Fig. 3a, BglI). However, because of the rapid replication of the HPV18E8^−^ genome, the frequencies of bidirectional theta replication intermediates and novel molecules were approximately equal 3 days post-transfection, as opposed to 5 days post-transfection for the replication of the HPV18wt genome (compare Fig. 2a to 3a, BglI).

As expected, we were able to identify the RIs arising via bidirectional theta replication (Fig. 3a). In the case of digestion with BglI and Bpu1102I, theta RIs appeared in the form of dY intermediates; in the case of digestion with PsyI and XmaJI, theta replication resulted in replication bubble intermediates (Fig. 3a, marked with black arrows). With both PsyI and XmaJI digestion, the signals representing the replication bubble intermediates became diffuse and tilted upward as the molecular weight of the bubble RIs neared 16 kbp, twice the size of the monomeric viral genome. These signals likely indicate the conversion of replication bubble intermediates into asymmetric dY intermediates because both digestions resulted in linear HPV18 genomes containing origins of replication in noncentral positions (Fig. 3c).

In the case of BglI digestion, there was a clear accumulation of molecules with large molecular masses and branched shapes; however, in the cases of Bpu1102I, PsyI and XmaJI digestions, the migration patterns of large and branched molecules were notably different from that of the BglI digestion (Fig. 3a, marked by black arrowheads). With PsyI and XmaJI digestions, the late theta RIs were expected to contain severely asymmetric dY molecules (as opposed to symmetric dY molecules generated via BglI digestion), explaining the different migration patterns. However, this does not apply to the Bpu1102I digestion, indicating that several populations of large and branched molecules of different origin migrated together to form the accumulated signal.

In addition to the RIs that arose through bidirectional theta replication, there was a strong presence of novel molecules, which retained the same migration pattern in all four restriction enzyme digestions (Fig. 3a, marked with white arrows). To analyze whether these novel molecules might represent molecules undergoing replication, XmaJI- and Bpu1102I digested HPV18E8^−^ LMW DNA was analyzed via 3D N/N/A AGE (Fig. 3b). 3D N/N/A AGE involves soaking a 2D N/N gel in alkali to denature DNA, followed by running a third dimension AGE to separate nascent and parental strands. The LMW DNA used in the 3D N/N/A AGE was extracted from HPV18E8^−^-transfected U2OS cells 5 days post-transfection, when the novel molecules were more prevalent than theta RIs. The migration patterns of parental and nascent strands originating from different RIs and parental strands originating from non-replicating molecules (represented by hemicatenanes) during 3D N/N/A analyses are depicted in Fig. 3d 41.

Following denaturation, the novel molecules separated into parental (Fig. 3b, P) and nascent (Fig. 3b, N) strands, demonstrating that these molecules are intermediates of replication. The origin of the nascent strands was confirmed by running the third dimension AGE in two different directions (data not shown). The nascent strands of the novel RIs traced a single arc containing molecules with continuously increasing length, eventually reaching the full length of the parental strands.

These results indicate that two different groups of RIs arise during the initial amplification of the HPV18 genome. One group of RIs arises via bidirectional theta replication initiated at the origin of replication situated in the non-coding region of the HPV18 genome. The second group of RIs displayed the same migration pattern during 2D N/N AGE, irrespective of the position of the cleavage site of the restriction enzyme used to digest the viral genome, suggesting that these secondary RIs are created via a mechanism that lacks specific origin sequences and can initiate replication in various regions of the HPV genome. Collectively, these results indicate essential differences between the two sets of RIs arising during the initial amplification of HPV18 genomes in U2OS cells, demonstrating the involvement of two separate replication mechanisms in the replication of the HPV18 genome.

### Nascent strands arising via the second replication mechanism are characteristic of unidirectional replication

**Figure 4:**
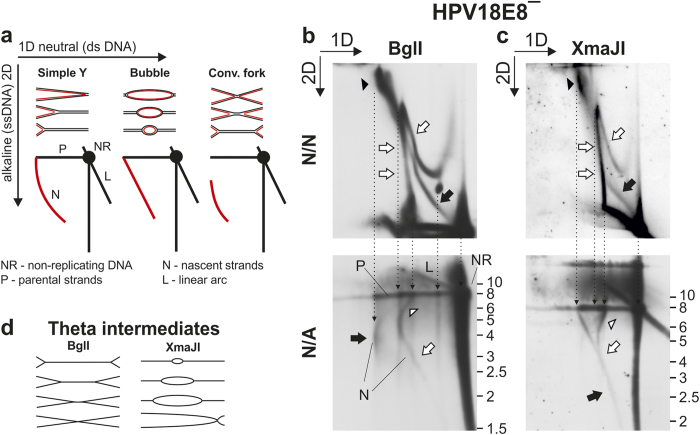
Analysis of nascent strands arising during the initial amplification of HPV18E8ˉ episomes via 2D N/A AGE analysis. Black arrowheads, putative late theta RIs; white arrowheads, irregularity in the migration pattern of the intermediates of the second replication mechanism; black arrows, theta RIs; white arrows, intermediates of the second replication mechanism. The direction of the gel electrophoresis in the first (1D) and second (2D) dimension of the N/N and N/A AGE is indicated in the top left corner of panels (b,c). The migration pattern of linear DNA ladder fragments during the second dimension of the N/A AGE is indicated on the right of the N/A figures. (**a**) A schematic depiction of the migration patterns formed by nascent strands originating from different types of RIs during 2D N/A AGE^42^. (**b**) Comparative 2D N/N and N/A AGE analysis of BglI-digested HPV18E8ˉ episomes extracted from U2OS cells 3 days post-transfection. (**c**) Comparative 2D N/N and N/A AGE analysis of XmaJI-digested HPV18E8ˉ episomes extracted from U2OS cells 3 days post-transfection. (**d**) A scheme depicting bidirectional theta RIs digested with BglI and XmaJI.

For a detailed analysis of the nascent strands arising during the initial amplification of the HPV18 genome, HPV18E8^−^ LMW DNA was linearized with BglI (Fig. 4b) or XmaJI (Fig. 4c) and analyzed comparatively via 2D N/N and N/A AGE. The first dimension of the 2D N/A AGE was run under neutral conditions; prior to running the second dimension, however, the gel was soaked in alkali to separate the parental and nascent strands. The migration patterns of the nascent strands originating from different types of RIs during 2D N/A AGE are depicted in Fig. 4a^42^. BglI- and XmaJI-digested RIs created during bidirectional theta replication are depicted in Fig. 4d. Because the first dimensions of the N/N and N/A AGE were performed under identical conditions, the results were aligned based on the migration progressions during the first dimension (Fig. 4b,c).

After digestion with BglI, theta replication resulted in dY intermediates (Fig. 4b, N/N, marked by a black arrow). The dY intermediates contain nascent strands that are expected to form a characteristic disrupted arc during 2D N/A analysis (Fig. 4a), which can be clearly detected in Fig. 4b N/A (marked by a black arrow). In the case of XmaJI digestion, bidirectional theta replication resulted in replication bubble intermediates (Fig. 4c, N/N, marked by a black arrow). Nascent strands originating from replication bubble intermediates form a straight diagonal line during 2D N/A analysis (Fig. 4a), also present during the analysis of XmaJI-digested HPV18E8^−^ genomes (Fig. 4c, N/A, marked by a black arrow). The alignments clearly indicate that the large and branched RIs that accumulate during HPV18 replication (Fig. 4b,c, N/N, marked by black arrowheads) separate into nascent strands that are typical of RIs arising via bidirectional theta replication, confirming that these intermediates contain almost fully replicated, late theta RIs. This analysis does not elucidate the nature of additional molecules migrating together with the late theta intermediates.

The nascent strands arising from the intermediates of the second HPV replication mechanism (Fig. 4b,c, N/A, marked by a white arrow) formed a signal that was initially similar to the arc of nascent strands originating from the replication bubble intermediates (Fig. 4a). During the analysis of the XmaJI-digested HPV18E8^−^ genomes, the signals representing nascent strands originating from bidirectional theta and the second replication mechanism partially converge, which is a strong indication of shared characteristics (Fig. 4c, N/A, marked by black and white arrows). As secondary replication progresses, the elongating nascent strands acquired a migration pattern that shared characteristics with the migration pattern of simple Y or severely asymmetric dY molecules, eventually reaching the size of the parental strands (compare Fig. 4b,c, N/A with Fig. 4a). The conversion of the migration pattern of the nascent strands originating from the intermediates of the second replication mechanism (from resembling the nascent strands arising from replication bubble intermediates to resembling the nascent strands arising from simple Y/asymmetric dY RIs) combined with the nascent strands achieving the same length as parental strands is only possible when the secondary RIs contain a unidirectional replication fork. It is important to distinguish that although the secondary RIs contain nascent strands migrating similarly to nascent strands originating from the replication bubble and simple Y/asymmetric dY RIs, the intermediates of the second replication mechanism displayed a complex shape during 2D N/N AGE analysis that was not characteristic of the abovementioned RIs, suggesting the presence of additional structural elements. The arc of secondary RIs also displayed a curious bend toward the signal representing non-replicated molecules as the length of the nascent strands neared maximum size (Fig. 4b and c, N/A, marked by a white arrowhead), which is not characteristic of simple Y or asymmetric dY RIs (Fig. 4a). During the N/N analysis, secondary RIs migrating as 16-kbp molecules (Fig. 4b,c, N/N, marked by double white arrows) formed a tilted peak, with intermediates that had shapes more similar to linear molecules migrating closer to the non-replicated molecules during the first dimension of the analysis, and the extremely branched molecules displaying a more retarded migration pattern. The bend in the signal representing nascent strands synthesized via the second replication is therefore likely caused by structural differences influencing the migration pattern of the secondary RIs during the first dimension of the 2D AGE.

2D N/A AGE analysis confirmed that two groups of RIs arise during the initial amplification of the HPV18 genome, as nascent strands arising via bidirectional theta replication and the second replication mechanism were clearly distinguishable. This analysis also demonstrated that the intermediates of the second replication mechanism contain a unidirectional replication fork.

### Analysis of subgenomic HPV18E8^−^ fragments confirms the involvement of a unidirectional replication mechanism without specific origin sequences in the initial amplification of the HPV18 genome

**Figure 5:**
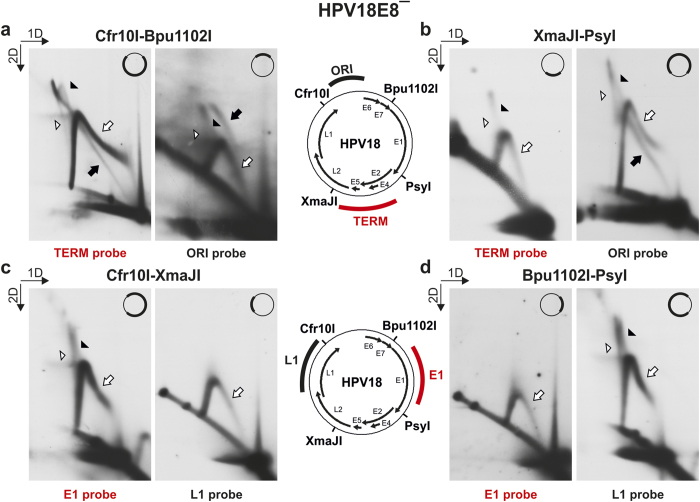
2D N/N AGE analysis of RIs arising from subgenomic fragments of HPV18E8ˉ episomes extracted from U2OS cells 3 days post-transfection. The positions of the recognition sites of used restriction endonucleases Cfr10I, Bpu1102I, PsyI and XmaJI are marked in both schemes. The HPV18 genomic areas specific for the ORI and TERM hybridization probes used for the analysis of Cfr10I-Bpu1102I and XmaJI-PsyI fragments (**a,b**) are marked in the upper scheme; the areas specific for the E1 and L1 probes used for the analysis of Cfr10I-XmaJI and Bpu1102I-PsyI fragments (**c,d**) are marked in the lower scheme. A simplified version of the schemes is in the upper right corner of each figure, with the analyzed fragment marked in bold. Black arrowheads, putative late theta RIs; white arrowheads, X-shaped molecules; black arrows, theta RIs; white arrows, intermediates of the second replication mechanism. The direction of the gel electrophoresis in the first (1D) and second (2D) dimension is indicated in the top left corners of the panels. (**a**) 2D N/N AGE of the Cfr10I-Bpu1102I fragments. (**b**) 2D N/N AGE of the XmaJI-PsyI fragments. (**c**) 2D N/N AGE of the Cfr10I-XmaJI fragments. (**d**) 2D N/N AGE of the Bpu1102I-PsyI fragments.

To confirm the results obtained during the analysis of nascent strands, a series of restriction analyses were performed with HPV18E8^−^ DNA extracted from U2OS cells 3 days post-transfection (Fig. 5). Different combinations of linearizing enzymes (Cfr10I, Bpu1102I, PsyI, XmaJI) were used to cleave the HPV18 genome into approximately 2-kbp and 6-kbp fragments, which were subsequently analyzed via 2D N/N AGE. Four distinct probes (the ORI, TERM, E1 and L1 probes) were used to selectively analyze the RIs arising from the fragments.

The larger, approximately 6-kbp fragments that contained either the origin of replication (Fig. 5b, XmaJI-PsyI ORI fragment) or the area where the progression of replication forks arising via theta replication was expected to terminate (Fig. 5a, Cfr10I-Bpu1102I TERM fragment) in a central position resulted in the expected theta RIs (the dY intermediates for the Cfr10I-Bpu1102I fragment and the bubble intermediates for the XmaJI-PsyI fragment, marked with black arrows). The large and branched RIs were present during the analysis of all 6-kbp fragments (Fig. 5, marked by black and white arrowheads). Reducing the size of the analyzed fragment improved the migration of these molecules to the extent that two different populations could be clearly distinguished. The first population (Fig. 5, marked by black arrowheads) had the characteristics of dY intermediates; the second population (Fig. 5, marked by white arrowheads), however, had a migration pattern traditionally associated with X-shaped molecules^43^. The appearance of the large and branched RIs during the analysis of the XmaJI-PsyI ORI fragment (Fig. 5b) could indicate that the replication forks arising via theta replication may converge anywhere from the end of the E1 ORF to the middle of the L2 ORF. Alternatively, the large and branched intermediates that were present in both the 6-kbp and 2-kbp fragments not containing the E4-E5 region of the HPV genome (Fig. 5a Cfr10I-Bpu1102I and Fig. 5b XmaJI-PsyI ORI fragments) may have arisen via presently unknown mechanisms not associated with bidirectional theta replication.

Most notably, all of the 6-kbp fragments contained the intermediates of the second replication mechanism (Fig. 5, marked with white arrows). The signals representing these intermediates were similar in their appearances regardless of the analyzed fragment and retained all of the characteristics that were evident during the analysis of the linearized HPV genomes (Fig. 3a, marked with white arrows). These results again suggest that the secondary replication mechanism does not utilize a specific initiation sequence.

The RIs that were present during the analysis of the small, approximately 2-kbp HPV18E8^−^ genomic fragments also displayed striking similarities (Fig. 5). The fragments that contained either the origin of replication (Fig. 5a, Cfr10I-Bpu1102I ORI fragment) or the area where theta replication forks are expected to converge (Fig. 5b, XmaJI-PsyI TERM fragment) in a central position resulted in the expected theta RIs (the bubble intermediates for the Cfr10I-Bpu1102I fragment and the dY intermediates for the XmaJI-PsyI fragment, marked with a black arrow and a black arrowhead, respectively). The lack of accumulation of dY molecules in the XmaJI-PsyI TERM fragment (Fig. 5b, compare with Fig. 3a BglI) indicates that the replication forks arising via bidirectional theta replication may converge over a wide area of the viral genome, and the termination of the progression of the replication forks is most likely not connected to any specific viral sequence.

Notably, no 2-kbp fragment contained molecules that resembled the secondary RIs that were present during the analysis of the 6-kbp fragment, but all contained the simple Y intermediates (Fig. 5, marked with white arrows). The simple Y intermediates must therefore also include molecules that arise via the second replication mechanism, supporting the data obtained during the 2D N/A AGE analyses suggesting that the second replication mechanism is unidirectional. The presence of simple Y intermediates in all analyzed 2-kbp fragments also confirms that the second HPV replication mechanism is without specific origin and termination sequences.

### The second replication mechanism can initiate unidirectional replication forks in both directions

**Figure 6:**
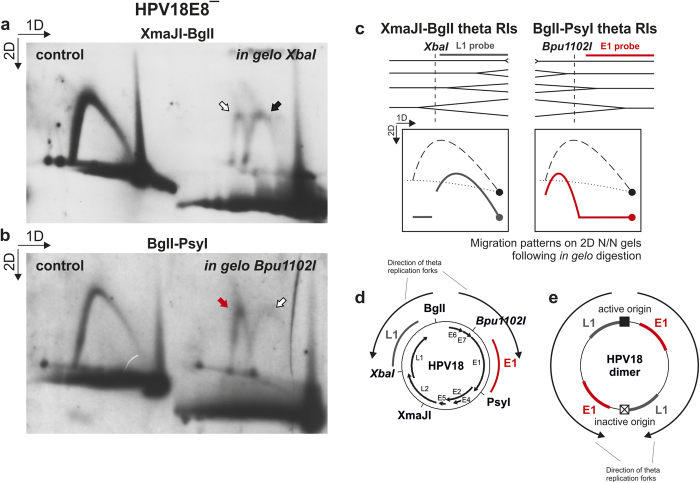
*In gelo* restriction analysis of HPV18E8ˉ episomes. The direction of the gel electrophoresis in the first (1D) and second (2D) dimension is indicated in the top left corner of panels a and b. Black or red arrow, replication forks arising via bidirectional theta replication; white arrows, replication forks travelling in the opposite direction of theta replication forks. (**a**) *In gelo* restriction analysis of the XmaJI-BglI fragment of the HPV18E8ˉ genomes extracted from U2OS cells 3 days post-transfection. XbaI was used for *in gelo* digestion, and the XmaJI-BglI fragment was selectively hybridized with an L1-specific probe. The control sample was analyzed under identical 2D N/N AGE and hybridization conditions but without the *in gelo* digestion step. (**b**) *In gelo* restriction analysis of the BglI-Psy fragment of the HPV18E8ˉ genomes extracted from U2OS cells 3 days post-transfection. The fragment was digested with Bpu1102I *in gelo* and selectively hybridized with a probe specific for the E1 region of the HPV18 genome. The control sample was analyzed under identical 2D N/N AGE and hybridization conditions but without the *in gelo* digestion step. (**c**) A schematic representation of theta RIs arising from the HPV18E8ˉ XmaJI-BglI and BglI-PsyI fragments and their expected migration pattern during 2D N/N AGE following *in gelo* digestion with the XbaI or Bpu1102I enzymes^44^. (**d**) A schematic depiction of the HPV18 genome. The recognition sites of the restriction enzymes BglI, PsyI and XmaJI are marked in regular font; the recognition sites of the endonucleases used for *in gelo* digestion (XbaI and Bpu1102I) within the analyzed fragments are marked in italics. The HPV18 genomic areas specific for the E1 and L1 hybridization probes and the directions of the replication forks created by bidirectional theta replication are also depicted. (**e**) A schematic depiction of the bidirectional theta replication of a HPV18 dimeric genome with only one active origin of replication. The Bpu1102-PsyI fragment generated following *in gelo* digestion and detected with the E1 probe is marked with red, and the XbaI-BglI fragment arising via *in gelo* digestion and detected with the L1 probe is marked with grey.

To determine the polarity of replication forks initiated by the second replication mechanism, HPV18E8^−^ LMW DNA samples extracted 3 days post-transfection from U2OS cells were digested with either BglI and XmaJI (Fig. 6a) or BglI and PsyI (Fig. 6b) and analyzed via *in gelo* digestion. Control samples that were not submitted to *in gelo* digestion were included in the 2D N/N AGE analysis of both fragments (Fig. 6a,b).

As HPV18E8^−^ genomes undergo bidirectional theta replication initiated from the origin of replication situated in the non-coding region of the viral genome, both fragments are expected to contain replication forks moving away from the BglI restriction site and toward the PsyI and XmaJI restriction sites. The directions of replication forks arising via bidirectional theta replication are marked in Fig. 6d, the migration patterns of these replication forks following *in gelo* digestion are depicted in Fig. 6c 44. Replication forks arising via bidirectional theta replication can be observed in both XmaJI-BglI and BglI-PsyI fragments (Fig. 6a,b, marked with black and red arrows). In addition to replication forks arising from theta replication, however, replication forks moving in the opposite direction can also be observed in both fragments (Fig. 6a,b, marked with white arrows). Bidirectional theta replication of dimeric HPV18 molecules with only one active origin of replication would result in replication forks appearing to move in opposite directions, but actually originating from the same initiation event (Fig. 6e). During the bidirectional theta replication of dimeric molecules, the ratio of the replication forks travelling through the XmaJI-BglI and BglI-PsyI fragments in opposite directions is equal. However, this does not apply to the results obtained during the analysis of the BglI-PsyI fragment (Fig. 6b). Additionally, the analysis of the linearized HPV genomes did not indicate the presence of theta RIs arising from such dimeric genomes (Figs 2a, 3a). It is therefore likely that the analyzed replication forks represent intermediates of the second replication mechanism, indicating that the second replication mechanism is capable of initiating unidirectional replication forks in both directions.

### Initial amplification of the HPV18 genome in HaCaT cells proceeds via two replication mechanisms

**Figure 7:**
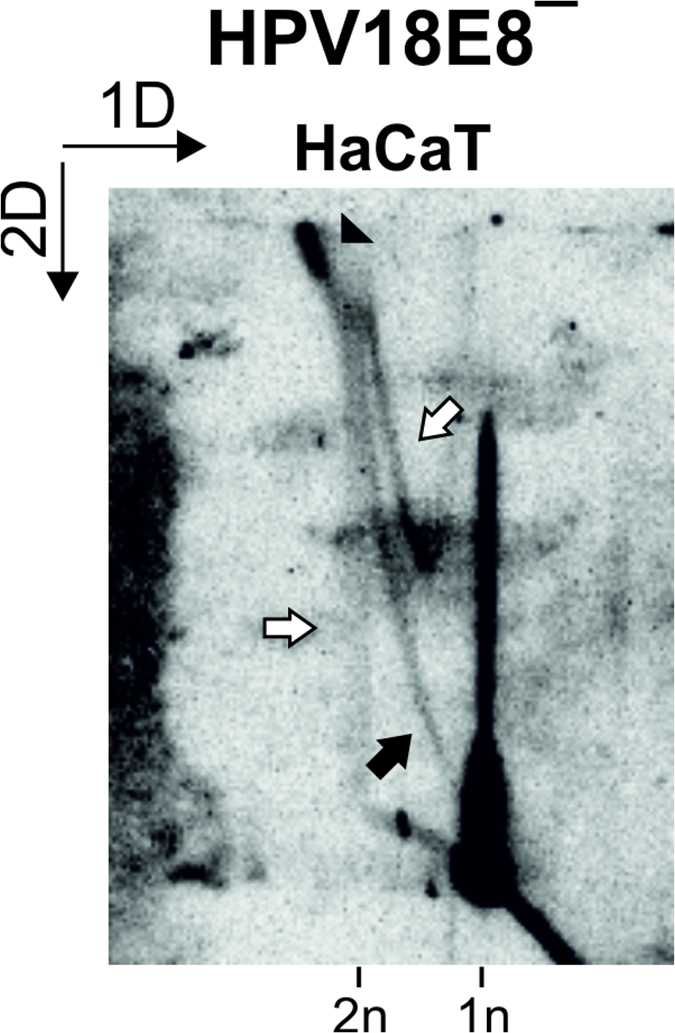
Analysis of RIs arising during the initial amplification replication of HPV18E8ˉ episomes in HaCaT cells. LMW DNA was extracted from HaCaT cells 5 days post-transfection, digested with BglI and analyzed via 2D N/N AGE. Black arrowhead, putative late theta RIs; black arrow, theta RIs; white arrows, intermediates of the second replication mechanism. 1n, monomeric (8-kbp) linear molecules; 2n, dimeric (16-kbp) linear molecules. The direction of the gel electrophoresis in the first (1D) and second (2D) dimension is indicated in the top left corner.

To exclude the possibility that the second replication mechanism was specific to the initial amplification of the HPV18 genome in U2OS cells, BglI-digested LMW DNA samples extracted from HaCaT cells 5 days post-transfection were analyzed via 2D N/N AGE (Fig. 7). The HPV-negative HaCaT cell line was established from spontaneously immortalized human keratinocytes and has retained full epidermal differentiation capacity^45^. HaCaT cells are capable of supporting HPV18 genomic replication, although at a significantly lower efficiency than U2OS cells (Fig. 7). Crucially, the dY molecules representing the intermediates of bidirectional theta replication (Fig. 7, marked with a black arrow) and the intermediates of the second replication mechanism (Fig. 7, marked with white arrows) are present. The accumulation of large and branched molecules can also be noted in HaCaT cells (Fig. 7, marked with a black arrowhead). These results demonstrate that the bidirectional theta replication and the secondary replication mechanism are not limited to the initial amplification replication of the HPV18 genome in the U2OS cell line alone.

## Discussion

Determining the mechanism behind HPV genomic replication is an essential prerequisite for understanding the viral life cycle and for developing efficient and specific drugs against HPV infection. The viral and cellular factors required for the initiation of the first amplification phase of papillomavirus replication have been extensively studied^8^,^9^,^10^,^11^,^12^,^13^,^14^,^15^; however, to date, no one has been able to study the RIs arising during the initial phase of HPV replication or the mechanism behind HPV replication. The development of the U2OS cell line-based assay^19^ has enabled the study of the early events in genomic HPV replication. Using the U2OS assay system, we determined that the initial amplification replication of circular extrachromosomal HPV18 genomes proceeds via bidirectional theta structures initiated from the previously described origin of replication situated in the non-coding region of the viral genome (Figs 1a, 2a, 3a, 4). We also noted a considerable accumulation of large and branched molecules containing almost fully replicated, late intermediates of bidirectional theta replication (Figs 2a, 3a, 4), indicating that the virus may have difficulties with completing theta replication. The accumulation of late replication intermediates has previously been noted in the polyomavirus SV40^46^,^47^. SV40 replication forks are arrested when bidirectional replication is approximately 91% completed, likely to prepare for the separation of daughter molecules^47^,^48^. The accumulation of late replication intermediates was proposed to indicate that the separation of daughter molecules is a slow, rate-limiting process^47^. These accumulated molecules appear to be associated with molecules that display characteristics common to recombination intermediates (Fig. 5). Intermediates characteristic of bidirectional replication via theta structures were also present during the initial amplification replication of the HPV18 genome in HaCaT cells (Fig. 7).

We also observed the presence of RIs that were not created via bidirectional theta replication during the first amplification phase of HPV18 replication (Figs 2a, 3, 4, 5, 6, 7). A clear temporal difference was observed in the appearance of these secondary RIs relative to the presence of theta RIs (Fig. 2a), indicating that bidirectional theta replication is responsible for the initial multiplication of the viral genomes, and the onset of the second replication mechanism occurs later. It is important to note that the two mechanisms appear to function together without resulting in the creation of aberrant RIs, indicating that the switch between the two mechanisms is regulated.

The second replication mechanism does not appear to have any specific initiation sequence (Figs 3a, 5) and results in RIs with exceeding structural complexity (Figs 2a, 3a, 5). The analysis of the nascent strands arising from the secondary RIs via 2D N/A AGE and the subgenomic HPV18 fragments via 2D N/N AGE demonstrated that the second replication mechanism is unidirectional (Figs 4, 5). *In gelo* digestion analysis indicated that secondary replication can be initiated in both directions (Fig. 6). Intermediates of the second replication mechanism also arise during the initial replication of HPV18 genomes in the HaCaT cell line (Fig. 7). The migration pattern of the intermediates of the second replication mechanism during the 2D N/N AGE of uncut HPV18 RIs is unclear. Because the analysis of uncut circular molecules did not resolve structural differences as well as the analysis of linearized RIs, the secondary RIs may migrate in a pattern indistinguishable from the migration pattern of theta RIs. We consider it likely that the secondary RIs migrate as the signal appearing to emanate either from the 1oc or 2ccc HPV genomes (Fig. 1c, white arrow). Alternatively, the uncut secondary RIs may migrate as the diffuse RIs (Fig. 1c, thin arrow).

Notably, the molecular mass of the secondary replication DNA synthesis intermediates always starts from approximately 1.2–1.4n and never emanates from the signal representing linearized 1n non-replicating molecules. We may be unable to detect the initiation structures of secondary replication because they are extremely labile and prone to dissociation, or because the initiation intermediates of a certain molecular weight exist in various conformations, thus resulting in a signal too diffuse to detect. Alternatively, the initiation structures of the second replication mechanism may be larger and with more complex structures than linear 1n molecules.

Analysis of the stable maintenance replication of HPV genomes in cell lines derived from naturally infected cervical tissues (the W12 cell line and the CIN612 cell line) and the keratinocyte cell line NIKS indicated that HPV genomes are inherently capable of replicating via two different modes, either once-per-S-phase as cellular DNA or randomly, with the choice of the replication mode dictated by the host cell^49^. The availability of the viral replication protein E1 is crucial in determining the mode of replication, with high expression of E1 converting replication to random-choice mode^49^. In the case of HPV16, the E1 protein is dispensable for the stable maintenance replication of the viral genome; however, it is absolutely necessary for both the initial and vegetative amplification phases of viral replication^50^. The replication of HPV genomes in the W12 and CIN612 cell lines has also been shown to proceed via two different mechanisms^16^. During stable maintenance replication, bidirectional theta replication is predominant in both cell lines; however, when the vegetative amplification of HPV genomes was induced, viral replication switches to a unidirectional replication mode with no specific initiation or termination sequences^16^. The reported RIs that appeared during the vegetative amplification of HPV genomes in W12 and CIN612 cell lines shared several characteristics with the secondary RIs arising during the initial amplification of the HPV18 genome in U2OS cells described in the present paper. There are several indications of two different replication mechanisms being utilized for the replication of the HPV genome during the stable maintenance and vegetative amplification replication; however, we propose that HPV genomes are capable of using two different replication mechanisms during the initial amplification phase of replication. The first rounds of HPV replication likely proceed via bidirectional theta replication; however, the onset of the second replication mechanism is soon initiated. Some characteristics of the second replication mechanism (e.g. no apparent need for a specific replication initiation sequence) indicate that the mechanism may be controlled by host factors. It is therefore conceivable that HPV genomes may be capable of alternating between two different replication mechanisms depending on the conditions in the host cell.

One likely candidate for the second replication mechanism is cellular recombination-dependent replication. Evidence for the association of HPV replication with cellular DNA damage response (DDR) and recombination machinery has been gradually mounting^25^,^26^,^51^,^52^,^53^,^54^,^55^. Activation of DDR pathways and the recruitment of factors that are involved in homologous recombination are necessary for the late amplification of the HPV genomes^52^,^53^,^54^. Similarly, there are indications of the involvement of the cellular DDR and recombination machinery in the initial amplification of HPV genomes^25^,^26^. Therefore, the second replication mechanism may be associated with the cellular recombination machinery.

We propose that the second replication is initiated by the invasion of a resectioned ssDNA end originating from dsDNA breaks in the HPV18 genome into an intact homologous HPV molecule. The origin of the dsDNA breaks is unclear, but they may arise during bidirectional theta replication because of nicks in parental strands or collapsed replication forks. The invasion of the resectioned ssDNA end results in the creation of D-loop structures and eventually the assembly of a unidirectional replication fork, which proceeds to duplicate the HPV genome. It must be noted that the analysis of uncut HPV18 RIs gave no indication of the presence of sigma-shaped RIs (Fig. 1c), excluding the presence of recombination-dependent replication mechanisms resulting in molecules with long linear tails.

Several DNA viruses interact with the components of the cellular DDR. Polyomaviruses, a group of small tumorigenic DNA viruses similar to HPV, require the activation of DDR for efficient viral replication^56^,^57^,^58^. DDR activation has been proposed to be caused by viral replication^59^,^60^ and contribute to the maintenance of replication fork integrity during replication^59^. There are no reported indications that polyomaviruses would deploy recombination to replicate the viral genome. However, our group has previously demonstrated the replication-dependent creation of oligomeric HPV genomes through homologous recombination^26^, indicating a link between HPV replication and homologous recombination.

Our results demonstrate that a second replication mechanism is involved in HPV replication during the initial viral genome amplification, thereby shedding new light on the events that occur during the different phases of papillomavirus replication. Because the RIs created during the initial amplification replication of HPV genomes in U2OS cells were also present in the human keratinocyte cell line HaCaT, these results also serve as proof-of-concept for the suitability of the U2OS cell line-based assay system for the study of the replication mechanisms of HPV.

## Materials and Methods

### Cell lines and transfection

U2OS and HaCaT cells were grown in Iscove’s modified Dulbecco’s medium (IMDM) supplemented with 10% fetal calf serum, 100 U/ml penicillin and 0.1 mg/ml streptomycin. U2OS cells were transfected with 1 μg of HPV18 wild-type or E8^−^ minicircle genome through electroporation^8^ using a Bio-Rad Gene Pulser XCell apparatus supplied with a capacitance extender (Bio-Rad Laboratories) at 220 V, and capacitance set to 975 μF. HaCaT cells were transfected with 5 μg of HPV18E8^−^ minicircle genome and 50 μg of fish sperm carrier DNA (Amresco) through electroporation using the same equipment at 210 V, and capacitance set to 975 μF.

### Plasmids

The generation of the HPV18 E8 mutant genome has been previously described^24^. Both the HPV18wt and the E8 mutant were produced as covalently closed minicircle plasmids^29^. The pMC.BESPX minicircle production vector (containing the ColE1 origin of replication, kanamycin resistance gene, ΦC31 integrase attachment sites attB and attP separated by a multiple cloning site, and 32 consecutive I-SceI recognition sites) was inserted into the HPV18wt and E8 mutant genomes after nucleotide 7473, resulting in the generation of pMC.BESPX-HPV18 plasmids containing the full-length HPV18 genome between the attB and attP attachment sites. The pMC.BESPX-HPV18 plasmids were used to transform *E. coli* strain ZYCY10P3S2T, which contains genes expressing the ΦC31 integrase and the I-SceI homing endonuclease under the control of the araCBAD system. The transformed bacterial cells were grown in TB medium (Difco) at 37 °C until OD_600_ 5 was reached, after which an equal volume of minicircle induction mix (0.04 N NaOH and 0.02% l-arabinose in LB medium) was added to the culture to induce the expression of the ΦC31 integrase and the I-SceI endonuclease. The culture was incubated for an additional 5-8 hours at 32 °C to facilitate the ΦC31 integrase-mediated removal of the pMC.BESPX vector sequence from the HPV18 genome and subsequent degradation of the pMC.BESPX plasmid by bacterial exonucleases following linearization with the I-SceI endonuclease. HPV18 genomes were purified from bacterial cells in the form of covalently closed minicircle plasmids using the NucleoBond PC 500 EF kit (Macherey-Nagel).

### DNA extraction

LMW DNA containing extrachromosomal viral genomes was extracted from HPV18-transfected U2OS cells via the modified Hirt method^30^.

### 2D N/N AGE

The principles of 2D N/N AGE have been discussed previously^39^. The electrophoresis for the first dimension was run using a 0.4% agarose gel submerged in 0.5× Tris-borate-EDTA (TBE) buffer at 0.3 V/cm for 48 h at room temperature. No ethidium bromide was added to the gel or the buffer. During the first dimension, the molecules are separated primarily based on molecular weight. The electrophoresis for the second dimension was run perpendicular to the first dimension using a 1% agarose gel in 0.5x TBE buffer at 6 V/cm for 5–8 h at 4 °C. Using 0.5x TBE buffer instead of the customary 1xTBE or 1xTris-acetate-EDTA (TAE) buffer yielded the best results under our experimental conditions, and the modified 2D N/N AGE protocol has been previously used for the analysis of papillomavirus RIs^17^. Changing the buffer conditions did not significantly alter the migration pattern of any RIs (Supplementary Fig. 1S). For the comparative 2D N/N and 2D N/A AGE analysis (Fig. 4), the second dimension of the 2D N/N AGE analysis was run using a 0.8% agarose gel in 0.5× TBE buffer. Ethidium bromide was added to the gel and the buffer to a final concentration of 0.3 μg/ml. During the second dimension, the molecules are separated primarily based on structural conformation.

### 2D N/A AGE

The principles of 2D N/A AGE have been described previously^42^. The first dimension was similar to the 2D N/N AGE, using a 0.4% agarose gel run in 0.5x TBE buffer at 0.3 V for 48 h at room temperature without added ethidium bromide. During the first dimension, the molecules are separated primarily based on molecular weight. For the second dimension, an 0.8% agarose gel was prepared in ddH_2_O and soaked in alkaline electrophoresis buffer (40 mM NaOH, 2 mM EDTA) for 90 min upon solidification. The second dimension was run perpendicular to the first dimension in the alkaline electrophoresis buffer at 1.5 V/cm for 24 h at 4 °C. During the second dimension, the nascent strands are separated from the parental strands.

### 3D N/N/A AGE

The principles of the 3D N/N/A AGE have been discussed previously^41^. The first and second dimension gels were run identically to the 2D N/N AGE analysis described above. After the second dimension, the entire gel was soaked in alkaline electrophoresis buffer (40 mM NaOH, 2 mM EDTA) for 120 minutes. The third dimension was run in the same direction as the first dimension at 1 V/cm for 14–16 h at 4 °C.

### Southern transfer and hybridization

The capillary transfer of DNA from agarose gels to nylon transfer membranes and the subsequent hybridization of the membranes with radioactively labeled probes have been thoroughly described previously^19^,^26^. Hybridization probes were prepared from HPV genomic fragments using the DecaLabel DNA labeling kit (Thermo Scientific) and radioactive [α-^32^P]dCTP (PerkinElmer/Hartmann Analytic). The HPV18 genomic probe was used in experiments displayed in Figs 1, 2, 3, 4 and 7 and constitutes the entire HPV18 wild-type genome linearized with EcoRI. For the analysis of subgenomic fragments (Fig. 5), the following probes were used: the ORI probe (spans nucleotides 7048-12); the E1 probe (spans nucleotides 778-1771); the L1 probe (spans nucleotides 5554-6114); and the TERM probe (spans nucleotides 2681-4235). For the *in gelo* digestion analysis, E1 (spans nucleotides 1357-2779) and L1 (spans nucleotides 5776-7278) probes were used.

### *In gelo* digestion

The principles of *in gelo* digestion have been described previously^44^. Prior to analysis, HPV18 LMW DNA samples were digested with either BglI and PsyI or BglI and XmaJI. The first dimension AGE was run using a 0.4% agarose gel in 0.5x TBE buffer without ethidium bromide at 0.8 V/cm for 22 h at room temperature. The agarose lanes containing the samples were excised from the gel and washed with TE 10:0.1 (10 mM Tris pH 8.0, 0.1 mM EDTA) twice for 30 minutes. The gel slices were subsequently incubated in the appropriate restriction buffer (33 mM Tris-acetate pH 7.9, 10 mM Mg-acetate, 66 mM K-acetate and 0.1 mg/ml BSA) twice for 1 h. Following incubation, the gel slices were removed from buffer and wiped dry, and 60 μl of enzyme solution containing 200 units of the appropriate enzyme (Bpu1102I for the BglI-PsyI fragment and XbaI for the BglI-XmaJI fragment, both manufactured by Thermo Scientific) was applied to the slices. After adding the restriction enzyme, the slices were incubated at 37 °C for at least 2 h. The enzyme solution was added three times in total. The second dimension was run using a 1.6% agarose gel in 0.5x TBE buffer at 6 V/cm for 5 hours at 4 °C. Ethidium bromide was added to both the gel and the buffer to a final concentration of 0.3 μ/ml.

## Additional Information

**How to cite this article**: Orav, M. *et al.* Initial amplification of the HPV18 genome proceeds via two distinct replication mechanisms. *Sci. Rep.* **5**, 15952; doi: 10.1038/srep15952 (2015).

## Supplementary Material

Supplementary Information

## References

[b1] De Villiers E. M. Cross-roads in the classification of papillomaviruses. Virology 445, 2–10, 10.1016/j.virol.2013.04.023 (2013). 23683837

[b2] Lorincz A. T. *et al.* Human papillomavirus infection of the cervix: relative risk associations of 15 common anogenital types. Obstetrics and gynecology 79, 328–337 (1992). 10.1097/00006250-199203000-00002 1310805

[b3] Durst M., Gissmann L., Ikenberg H. & zur Hausen H. A papillomavirus DNA from a cervical carcinoma and its prevalence in cancer biopsy samples from different geographic regions. Proc Natl Acad Sci USA 80, 3812–3815 (1983). 10.1073/pnas.80.12.3812 PMC394142 6304740

[b4] Walboomers J. M. M. *et al.* Human papillomavirus is a necessary cause of invasive cervical cancer worldwide. Journal of Pathology 189, 12–19, 10.1002/(Sici)1096-9896(199909)189:1<12::Aid-Path431>3.0.Co;2-F(1999 ). 10451482

[b5] McLaughlin-Drubin M. E. & Munger K. Viruses associated with human cancer. Bba-Mol Basis Dis 1782, 127–150, 10.1016/j.bbadis.2007.12.005 (2008). PMC2267909 18201576

[b6] Gillison M. L. & Lowy D. R. A causal role for human papillomavirus in head and neck cancer. Lancet 363, 1488–1489, 10.1016/S0140-6736(04)16194-1 (2004). 15135592

[b7] Doorbar J. *et al.* The biology and life-cycle of human papillomaviruses. Vaccine 30, Suppl 5, F55–70, 10.1016/j.vaccine.2012.06.083 (2012). 23199966

[b8] Ustav M. & Stenlund A. Transient replication of BPV-1 requires two viral polypeptides encoded by the E1 and E2 open reading frames. The EMBO journal 10, 449–457 (1991). 10.1002/j.1460-2075.1991.tb07967.x PMC452666 1846806

[b9] Yang L., Li R., Mohr I. J., Clark R. & Botchan M. R. Activation of BPV-1 replication *in vitro* by the transcription factor E2. Nature 353, 628–632, 10.1038/353628a0 (1991). 1656277

[b10] Remm M., Brain R. & Jenkins J. R. The E2 binding sites determine the efficiency of replication for the origin of human papillomavirus type 18. Nucleic acids research 20, 6015–6021 (1992). 10.1093/nar/20.22.6015 PMC334468 1334259

[b11] Frattini M. G. & Laimins L. A. Binding of the human papillomavirus e1 origin-recognition protein is regulated through complex-formation with the E2 enhancer-binding protein. P Natl Acad Sci USA 91, 12398–12402, 10.1073/pnas.91.26.12398 (1994). PMC45445 7809048

[b12] Chiang C. M. *et al.* Viral E1 and E2 proteins support replication of homologous and heterologous papillomaviral origins. Proc Natl Acad Sci USA 89, 5799–5803 (1992). 10.1073/pnas.89.13.5799 PMC402105 1321423

[b13] Sverdrup F. & Khan S. A. 2 E2 Binding-Sites Alone Are Sufficient to Function as the Minimal Origin of Replication of Human Papillomavirus Type-18 DNA. Journal of virology 69, 1319–1323 (1995). 10.1128/jvi.69.2.1319-1323.1995 PMC188713 7815514

[b14] Melendy T., Sedman J. & Stenlund A. Cellular factors required for papillomavirus DNA replication. Journal of virology 69, 7857–7867 (1995). 10.1128/jvi.69.12.7857-7867.1995 PMC189730 7494298

[b15] Kuo S. R., Liu J. S., Broker T. R. & Chow L. T. Cell-free replication of the human papillomavirus DNA with homologous viral E1 and E2 proteins and human cell extracts. The Journal of biological chemistry 269, 24058–24065 (1994). 7523366

[b16] Flores E. R. & Lambert P. F. Evidence for a switch in the mode of human papillomavirus type 16 DNA replication during the viral life cycle. Journal of virology 71, 7167–7179 (1997). 10.1128/jvi.71.10.7167-7179.1997 PMC192056 9311789

[b17] Yang L. & Botchan M. Replication of bovine papillomavirus type 1 DNA initiates within an E2-responsive enhancer element. Journal of Virology 64, 5903–5911 (1990). 10.1128/jvi.64.12.5903-5911.1990 PMC248757 2173772

[b18] Auborn K. J., Little R. D., Platt T. H., Vaccariello M. A. & Schildkraut C. L. Replicative intermediates of human papillomavirus type 11 in laryngeal papillomas: site of replication initiation and direction of replication. Proc Natl Acad Sci USA 91, 7340–7344 (1994). 10.1073/pnas.91.15.7340 PMC44395 8041792

[b19] Geimanen J. *et al.* Development of a cellular assay system to study the genome replication of high- and low-risk mucosal and cutaneous human papillomaviruses. Journal of virology 85, 3315–3329, 10.1128/JVI.01985-10 (2011). PMC3067845 21248030

[b20] Stubenrauch F., Hummel M., Iftner T. & Laimins L. A. The E8E2C protein, a negative regulator of viral transcription and replication, is required for extrachromosomal maintenance of human papillomavirus type 31 in keratinocytes. Journal of virology 74, 1178–1186 (2000). 10.1128/jvi.74.3.1178-1186.2000 PMC111452 10627528

[b21] Sankovski E., Mannik A., Geimanen J., Ustav E. & Ustav M. Mapping of betapapillomavirus human papillomavirus 5 transcription and characterization of viral-genome replication function. Journal of virology 88, 961–973, 10.1128/JVI.01841-13 (2014). PMC3911660 24198410

[b22] Zobel T., Iftner T. & Stubenrauch F. The papillomavirus E8-E2C protein represses DNA replication from extrachromosomal origins. Molecular and cellular biology 23, 8352–8362 (2003). 10.1128/MCB.23.22.8352-8362.2003 PMC262328 14585992

[b23] Lace M. J., Anson J. R., Thomas G. S., Turek L. P. & Haugen T. H. The E8—E2 gene product of human papillomavirus type 16 represses early transcription and replication but is dispensable for viral plasmid persistence in keratinocytes. Journal of virology 82, 10841–10853, 10.1128/JVI.01481-08 (2008). PMC2573160 18753207

[b24] Kurg R., Uusen P., Vosa L. & Ustav M. Human papillomavirus E2 protein with single activation domain initiates HPV18 genome replication, but is not sufficient for long-term maintenance of virus genome. Virology 408, 159–166, 10.1016/j.virol.2010.09.010 (2010). 20940072

[b25] Reinson T. *et al.* Engagement of the ATR-Dependent DNA Damage Response at the Human Papillomavirus 18 Replication Centers during the Initial Amplification. Journal of virology 87, 951–964, 10.1128/JVI.01943-12 (2013). PMC3554080 23135710

[b26] Orav M. *et al.* Recombination-dependent oligomerization of human papillomavirus genomes upon transient DNA replication. Journal of virology 87, 12051–12068, 10.1128/JVI.01798-13 (2013). PMC3807893 23986589

[b27] Toots M. *et al.* The Transcription Map of Human Papillomavirus Type 18 during Genome Replication in U2OS Cells. Plos One 9, e116151, 10.1371/journal.pone.0116151 (2014). PMC4280167 25548925

[b28] Reinson T., Henno L., Toots M., Ustav M. Jr. & Ustav M. The Cell Cycle Timing of Human Papillomavirus DNA Replication. Plos One 10, e0131675, 10.1371/journal.pone.0131675 (2015). PMC4489393 26132923

[b29] Kay M. A., He C. Y. & Chen Z. Y. A robust system for production of minicircle DNA vectors. Nature Biotechnology 28, 1287–1289, 10.1038/nbt.1708 (2010). PMC4144359 21102455

[b30] Hirt B. Selective extraction of polyoma DNA from infected mouse cell cultures. Journal of Molecular Biology 26, 365–369 (1967). 10.1016/0022-2836(67)90307-5 4291934

[b31] Martin-Parras L. *et al.* Topological complexity of different populations of pBR322 as visualized by two-dimensional agarose gel electrophoresis. Nucleic Acids Research 26, 3424–3432 (1998). 10.1093/nar/26.14.3424 PMC147708 9649629

[b32] Boshart M. *et al.* A new type of papillomavirus DNA, its presence in genital cancer biopsies and in cell lines derived from cervical cancer. The EMBO Journal 3, 1151–1157 (1984). 10.1002/j.1460-2075.1984.tb01944.x PMC557488 6329740

[b33] Kristiansen E., Jenkins A. & Holm R. Coexistence of Episomal and Integrated Hpv16 DNA in Squamous-Cell Carcinoma of the Cervix. J Clin Pathol 47, 253–256, 10.1136/Jcp.47.3.253 (1994). PMC501906 7677803

[b34] Durst M., Kleinheinz A., Hotz M. & Gissmann L. The physical state of human papillomavirus type 16 DNA in benign and malignant genital tumours. The Journal of General Virology 66 (Pt **7**), 1515–1522 (1985). 10.1099/0022-1317-66-7-1515 2991428

[b35] Cullen A. P., Reid R., Campion M. & Lorincz A. T. Analysis of the physical state of different human papillomavirus DNAs in intraepithelial and invasive cervical neoplasm. Journal of Virology 65, 606–612 (1991). 10.1128/jvi.65.2.606-612.1991 PMC239798 1846186

[b36] Choo K. B. *et al.* Presence of Episomal and Integrated Human Papillomavirus DNA-Sequences in Cervical-Carcinoma. J Med Virol 21, 101–107, 10.1002/jmv.1890210202 (1987). 3029316

[b37] Alazawi W. *et al.* Changes in cervical keratinocyte gene expression associated with integration of human papillomavirus 16. Cancer Research 62, 6959–6965 (2002). 12460913

[b38] Hong S. & Laimins L. A. The JAK-STAT transcriptional regulator, STAT-5, activates the ATM DNA damage pathway to induce HPV 31 genome amplification upon epithelial differentiation. PLoS pathogens 9, e1003295, 10.1371/journal.ppat.1003295 (2013). PMC3616964 23593005

[b39] Brewer B. J. & Fangman W. L. The localization of replication origins on ARS plasmids in S. cerevisiae. Cell 51, 463–471 (1987). 10.1016/0092-8674(87)90642-8 2822257

[b40] Belanger K. G., Mirzayan C., Kreuzer H. E., Alberts B. M. & Kreuzer K. N. Two-dimensional gel analysis of rolling circle replication in the presence and absence of bacteriophage T4 primase. Nucleic Acids Research 24, 2166–2175 (1996). 10.1093/nar/24.11.2166 PMC145913 8668550

[b41] Garcia-Luis J. & Machin F. Mus81-Mms4 and Yen1 resolve a novel anaphase bridge formed by noncanonical Holliday junctions. Nat Commun 5, Artn 5652Doi 10.1038/Ncomms6652 (2014). 25466415

[b42] Nawotka K. A. & Huberman J. A. Two-Dimensional Gel-Electrophoretic Method for Mapping DNA Replicons. Molecular and cellular biology 8, 1408–1413 (1988). 10.1128/mcb.8.4.1408-1413.1988 PMC363297 2837639

[b43] Collins I. & Newlon C. S. Meiosis-specific formation of joint DNA molecules containing sequences from homologous chromosomes. Cell 76, 65–75 (1994). 10.1016/0092-8674(94)90173-2 8287480

[b44] Brewer B. J., Lockshon D. & Fangman W. L. The Arrest of Replication Forks in the Rdna of Yeast Occurs Independently of Transcription. Cell 71, 267–276, 10.1016/0092-8674(92)90355-G (1992). 1423594

[b45] Boukamp P. *et al.* Normal Keratinization in a Spontaneously Immortalized Aneuploid Human Keratinocyte Cell-Line. J Cell Biol 106, 761–771, 10.1083/jcb.106.3.761 (1988). PMC2115116 2450098

[b46] Tapper D. P. & DePamphilis M. L. Discontinuous DNA replication: accumulation of Simian virus 40 DNA at specific stages in its replication. Journal of Molecular Biology 120, 401–422 (1978). 10.1016/0022-2836(78)90427-8 206700

[b47] Seidman M. M. & Salzman N. P. Late replicative intermediates are accumulated during simian virus 40 DNA replication *in vivo* and *in vitro*. Journal of Virology 30, 600–609 (1979). 10.1128/jvi.30.2.600-609.1979 PMC353363 224218

[b48] Tapper D. P. & DePamphilis M. L. Preferred DNA sites are involved in the arrest and initiation of DNA synthesis during replication of SV40 DNA. Cell 22, 97–108 (1980). 10.1016/0092-8674(80)90158-0 6253085

[b49] Hoffmann R., Hirt B., Bechtold V., Beard P. & Raj K. Different modes of human papillomavirus DNA replication during maintenance. Journal of Virology 80, 4431–4439, 10.1128/Jvi.80.9.4431.2006 (2006). PMC1471999 16611903

[b50] Egawa N. *et al.* The E1 Protein of Human Papillomavirus Type 16 Is Dispensable for Maintenance Replication of the Viral Genome. Journal of virology 86, 3276–3283, 10.1128/Jvi.06450-11 (2012). PMC3302310 22238312

[b51] Kadaja M., Isok-Paas H., Laos T., Ustav E. & Ustav M. Mechanism of genomic instability in cells infected with the high-risk human papillomaviruses. PLoS Pathogens 5, e1000397, 10.1371/journal.ppat.1000397 (2009). PMC2666264 19390600

[b52] Moody C. A. & Laimins L. A. Human papillomaviruses activate the ATM DNA damage pathway for viral genome amplification upon differentiation. PLoS Pathogens 5, e1000605, 10.1371/journal.ppat.1000605 (2009). PMC2745661 19798429

[b53] Gillespie K. A., Mehta K. P., Laimins L. A. & Moody C. A. Human papillomaviruses recruit cellular DNA repair and homologous recombination factors to viral replication centers. Journal of Virology 86, 9520–9526, 10.1128/JVI.00247-12 (2012). PMC3416172 22740399

[b54] Anacker D. C., Gautam D., Gillespie K. A., Chappell W. H. & Moody C. A. Productive replication of human papillomavirus 31 requires DNA repair factor Nbs1. Journal of Virology 88, 8528–8544, 10.1128/JVI.00517-14 (2014). PMC4135936 24850735

[b55] Fradet-Turcotte A. *et al.* Nuclear accumulation of the papillomavirus E1 helicase blocks S-phase progression and triggers an ATM-dependent DNA damage response. Journal of Virology 85, 8996–9012, 10.1128/JVI.00542-11 (2011). PMC3165840 21734051

[b56] Dahl J., You J. & Benjamin T. L. Induction and utilization of an ATM signaling pathway by polyomavirus. Journal of Virology 79, 13007–13017, 10.1128/JVI.79.20.13007-13017.2005 (2005). PMC1235815 16189003

[b57] Shi Y. L., Dodson G. E., Rundell K. & Tibbetts R. S. Ataxia-telangiectasia-mutated (ATM) is a T-antigen kinase that controls SV40 viral replication *in vivo*. Journal of Biological Chemistry 280, 40195–40200, 10.1074/jbc.C500400200 (2005). 16221684

[b58] Tsang S. H., Wang X., Li J., Buck C. B. & You J. X. Host DNA Damage Response Factors Localize to Merkel Cell Polyomavirus DNA Replication Sites To Support Efficient Viral DNA Replication. Journal of Virology 88, 3285–3297, 10.1128/Jvi.03656-13 (2014). PMC3957940 24390338

[b59] Sowd G. A., Li N. Y. & Fanning E. ATM and ATR Activities Maintain Replication Fork Integrity during SV40 Chromatin Replication. PLoS Pathogens 9, ARTN e100328310.1371/journal.ppat.1003283 (2013). 10.1371/journal.ppat.1003283 PMC3617017 23592994

[b60] Verhalen B., Justice J. L., Imperiale M. J. & Jiang M. X. Viral DNA Replication-Dependent DNA Damage Response Activation during BK Polyomavirus Infection. Journal of Virology 89, 5032–5039, 10.1128/Jvi.03650-14 (2015). PMC4403456 25694603

