# Supplementary material for: Initial amplification of the HPV18 genome proceeds via two distinct replication mechanisms
Source: Sci Rep. 2015 Nov 2;5:15952. doi: 10.1038/srep15952 (PMC4629122; doi:10.1038/srep15952)
Supplement: Supplementary Information [file srep15952-s1.pdf]

Supplementary Information

Initial amplification of the HPV18 genome proceeds via two distinct replication mechanisms

Marit Orav[1], Jelizaveta Geimanen[1], Eva-Maria Sepp[1], Liisi Henno[1], Ene Ustav[1,2] and Mart Ustav#[1,3,4]

[1] University of Tartu, Institute of Technology, Nooruse 1, 50411 Tartu, Estonia; [2] Estonian Biocentre, Riia 23, 51010 Tartu, Estonia; [3] Icosagen Cell Factory OÜ, Nooruse 9, 50411 Tartu, Estonia; [4] Estonian Academy of Sciences, Kohtu 6, 10130 Tallinn, Estonia.
# Corresponding author. Institute of Technology, University of Tartu; Nooruse 1, 50411, Tartu, Estonia.
Tel.: +3727375040; Fax: +3727374900; e-mail: mart.ustav@ut.ee

Supplementary Figure 1S

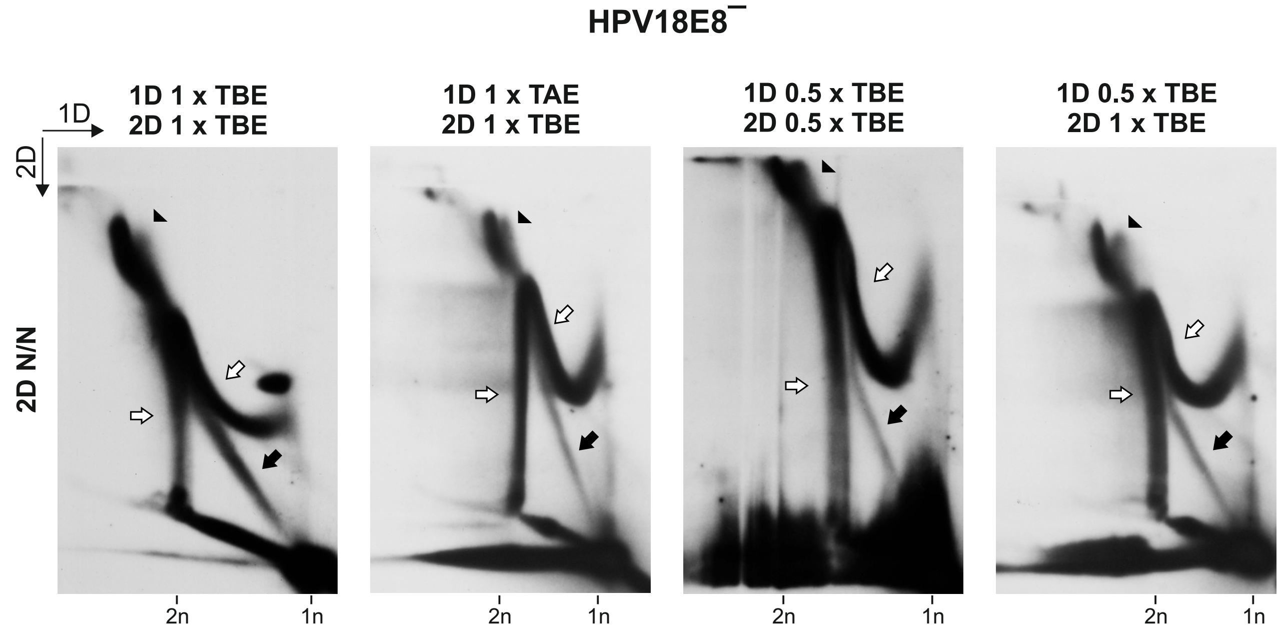

Figure 1SM. 2D N/N AGE analysis of BglI-digested HPV18E8¯ genomes. HPV18E8¯ LMW DNA was extracted from U2OS cells 3 days post-transfection and linearized with BglI. The samples were analyzed via 2D N/N AGE using 0.4% agarose gels run for 21h at 1V/cm for the first dimension and 1% agarose gels run for 6.5h at 6V/cm for the second dimension. The buffer conditions used during the first (1D) and second (2D) dimension are marked above each figure; the direction of the gel electrophoresis in the first (1D) and second (2D) dimension is indicated in the top left corner of the panel. When the second dimension was run in 1 x TBE buffer, buffer recirculation was used during the second dimension. 1lin, monomeric (8-kbp) linear molecules; 2lin, dimeric (16-kbp) linear molecules; black arrows, bidierctional theta replication intermediates; white arrows, non-theta replication intermediates; black arrowheads, putative late theta replication intermediates.
